# NaCl-Induced Elicitation Alters Physiology and Increases Accumulation of Phenolic Compounds in *Melissa officinalis* L.

**DOI:** 10.3390/ijms22136844

**Published:** 2021-06-25

**Authors:** Barbara Hawrylak-Nowak, Sławomir Dresler, Maria Stasińska-Jakubas, Magdalena Wójciak, Ireneusz Sowa, Renata Matraszek-Gawron

**Affiliations:** 1 Department of Botany and Plant Physiology, Faculty of Environmental Biology, University of Life Sciences in Lublin, Akademicka 15, 20-950 Lublin, Poland; jakubas.ms@gmail.com (M.S.-J.); renata.matraszek@up.lublin.pl (R.M.-G.); 2 Department of Analytical Chemistry, Medical University of Lublin, Chodźki 4a, 20-093 Lublin, Poland; slawomir.dresler@umlub.pl (S.D.); magdalenawojciak@umlub.pl (M.W.); i.sowa@umlub.pl (I.S.)

**Keywords:** phenolic metabolites, lemon balm, chlorophyll fluorescence, medicinal plants, secondary metabolites, abiotic elicitors, salinity

## Abstract

In nature, plants usually produce secondary metabolites as a defense mechanism against environmental stresses. Different stresses determine the chemical diversity of plant-specialized metabolism products. In this study, we applied an abiotic elicitor, i.e., NaCl, to enhance the biosynthesis and accumulation of phenolic secondary metabolites in *Melissa officinalis* L. Plants were subjected to salt stress treatment by application of NaCl solutions (0, 50, or 100 mM) to the pots. Generally, the NaCl treatments were found to inhibit the growth of plants, simultaneously enhancing the accumulation of phenolic compounds (total phenolics, soluble flavonols, anthocyanins, phenolic acids), especially at 100 mM NaCl. However, the salt stress did not disturb the accumulation of photosynthetic pigments and proper functioning of the PS II photosystem. Therefore, the proposed method of elicitation represents a convenient alternative to cell suspension or hydroponic techniques as it is easier and cheaper with simple application in lemon balm pot cultivation. The improvement of lemon balm quality by NaCl elicitation can potentially increase the level of health-promoting phytochemicals and the bioactivity of low-processed herbal products.

## 1. Introduction

Due to their sedentary lifestyle, plants are under constant pressure to adjust their metabolic pathways to changing environmental conditions. Therefore, in addition to primary metabolites, they synthesize a wide range of unique, low-molecular secondary metabolites. Environmental stresses (biotic and abiotic) redirect plant metabolism towards the biosynthesis of these metabolites from primary metabolites and intermediates. The products of specialized metabolism most often have defense and signaling functions. Such compounds are generally toxic or inconsumable for herbivores, possess fungicidal or bactericidal properties, or can detoxify toxic metals and consequently protect plants against stresses [1,2,3].

Therefore, stress is an important factor determining the chemical composition of plants and thus having a significant impact on their biological activity. The use of plant defense mechanisms to stimulate the biosynthesis of desired secondary metabolites and improve the health-promoting quality of plants is called elicitation [4]. Elicitors are physical factors or chemical substances that can induce responses via modifications of accumulation and/or synthesis of secondary metabolites, mimicking a defensive reaction [5,6,7]. One of the readily available and cheap abiotic elicitors with proven effectiveness is NaCl [8,9].

Salinity induces both ionic and osmotic stress in plants. The intensity of the salt stress plays a decisive role in the plant salinity response and the possibility of reversible or irreversible changes in plant functioning [10]. The effect of salt stress on plants can be twofold. Excess of salt may have a toxic effect on plants, limiting their growth and development, and may lead to plant death in extreme cases [11]. Nevertheless, a moderate or mild level of salinity may have a stimulating effect on the growth and development of plants (eustress) and/or the accumulation of secondary metabolites, improving the level of pro-health components, antioxidant potential, and nutritional quality of plants [5,8,12]. For example, in safflower (*Carthamus tinctorius* L.) grown at a salinity concentration <100 mM NaCl in hydroponic conditions, the accumulation of flavonoids was enhanced, but the plant growth was not reduced [13]. In turn, Navarro et al. [14] demonstrated improved antioxidant activity in hydrophilic and lipophilic fractions under moderate salinity in red pepper.

Lemon balm (*Melissa officinalis* L.), belonging to the family Lamiaceae, is a valuable herb used as a flavoring agent in food and drinks. Due to the richness of secondary metabolites (mainly monoterpenoids in essential oils and phenolic compounds), it is also used as a medicinal plant, natural insecticide, and an ingredient of cosmetics [15]. The many biologically active components in lemon balm include a number of phenolics, the most important of which is rosmarinic acid [16,17]. Ingredients of extracts from *M*. *officinalis* revealed a number of pharmacological activities, including clinically proven antiviral, anxiolytic, and antispasmodic effects as well as influence on mood stabilization and memory [18]. Due to the positive effect on cognitive function and agitation, *M*. *officinalis* extract is valuable in the management of mild to moderate Alzheimer’s disease [19]. This species is resistant to relatively low concentrations of salinity (up to 50 mM NaCl) [20]. Irrigation of lemon balm with saline water increased the essential oil yield, free proline, and total soluble sugar levels but decreased plant growth parameters [21]. In turn, a decrease in the yield of essential oils but an increase in the number of their ingredients under NaCl exposure (50–200 mM NaCl) was found by Bonacina et al. [20]. However, the effect of salinity on the level and composition of phenolic compounds in this species is poorly understood. One of the few studies on this topic indicated that NaCl-treated lemon balm accumulated more total phenolics and flavonoids than untreated plants [22].

The hypothesis that NaCl-induced stress affects physiological response and enhances accumulation of (poly)phenolics in lemon balm was tested. This elicitation method may potentially improve the level of health-promoting secondary metabolites and bioactivity of lemon balm-based raw materials. Moreover, this work provides insights into the effect of salt stress on the physiology and phenolic composition of the pharmaceutically significant species *M*. *officinalis*.

## 2. Results

### 2.1. Biomass and Physiological Parameters of Lemon Balm Grown under NaCl Exposure

**Figure 1:**
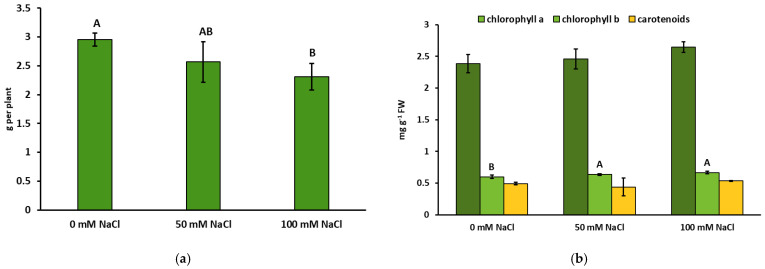
Effect of the NaCl concentration on (**a**) growth parameters and (**b**) concentrations of photosynthetic pigments in *Melissa officinalis* after 10 days of salt exposure. Data are means ± SD (*n* = 16 for FW and *n* = 6 for photosynthetic pigments). Means followed by different letters differ statistically significantly (*p* < 0.05, Tukey’s test). The absence of letters indicates that there are no significant differences.

The use of the NaCl solution for plant irrigation had a significant impact on the plant biomass (Figure 1a). A decrease in the FW of the above-ground organs with the increase in NaCl concentration was observed; however, this reduction (22% in relation to the control) was statistically significant only under the influence of 100 mM NaCl. In comparison to the control plants, an increase (by 6–11%) in the concentration of chlorophyll *b* at 50 and 100 mM NaCl was found. However, the level of chlorophyll *a* and carotenoids did not change significantly under the salt exposure (Figure 1b). The measurements of selected parameters of chlorophyll *a* fluorescence (F_0_, F_m_, and F_v_/F_m_) indicated that the applied concentrations of NaCl had no significant effects on the efficiency of photosynthesis (Supplementary Figure S1).

### 2.2. Total Phenolic Compounds, Flavonoids, Rosmarinic Acid, and Anthocyanin Concentrations under NaCl Elicitation

**Figure 2:**
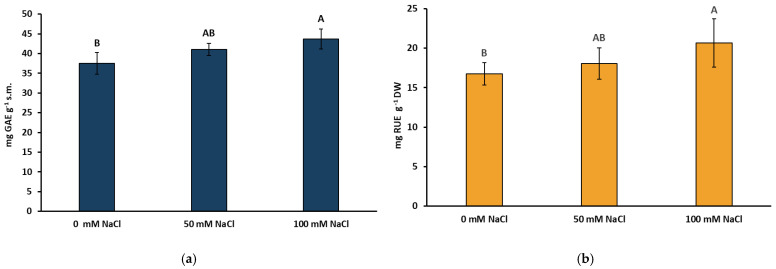
Effect of the NaCl concentration on the concentration of (**a**) total soluble phenolic compounds and (**b**) total soluble flavonols in *Melissa officinalis* shoots after 10 days of salt exposure. Data are means ± SD (*n* = 4). Means followed by different letters differ statistically significantly (*p* < 0.05, Tukey’s test).

Salinity influenced the total concentration of phenolic compounds (TPC) in the extracts made from the lemon balm herb. It was found that both of the different NaCl levels enhanced the TPC concentration, but this increase (by 16% compared to the control) was statistically significant only after the application of 100 mM NaCl (Figure 2a). Similarly, the content of total flavonol compounds (TFC) significantly increased by 23% after application of 100 mM NaCl (Figure 2b).

**Figure 3:**
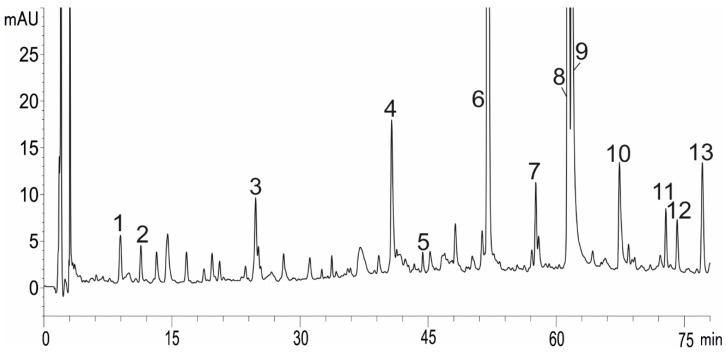
Chromatogram of *Melissa officinalis* leaf extract (control plants) with peaks identified by HPLC-UV-MS. (1) 3-(3,4-dihydroxyphenyl)-lactic acid; (2) caftaric acid; (3) fertaric acid; (4) caftaric acid hexoside; (5) rosmarinic acid hexoside; (6) rosmarinic acid; (7) salvianolic acid H/I (isomer); (8) salvianolic acid C derivative III; (9) lithospheric acid; (10) salvianolic acid C derivative III; (11) salvianolic acid C derivative IV; (12) sagecoumarin 2-hydroxy-3-(3,4-dihydroxyphenyl)-propanoide; and (13) methyl lithospermic acid.

**Figure 4:**
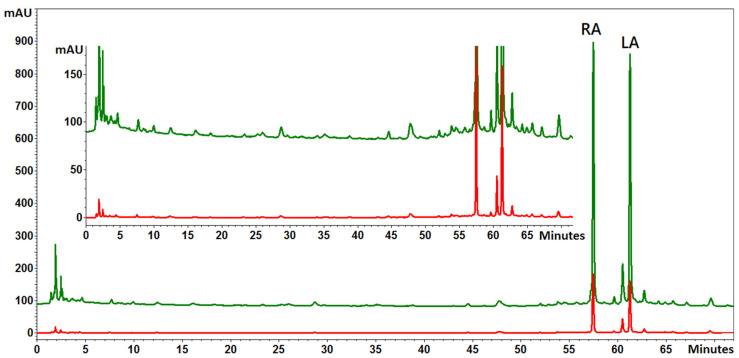
Comparison of chromatograms of *Melissa officinalis* leaf extracts (evaluated by HPLC-UV-MS) from control plants (red line) and from plants treated with 100 mM NaCl (green line); RA, rosmarinic acid; LA, lithospheric acid.

**Figure 5:**
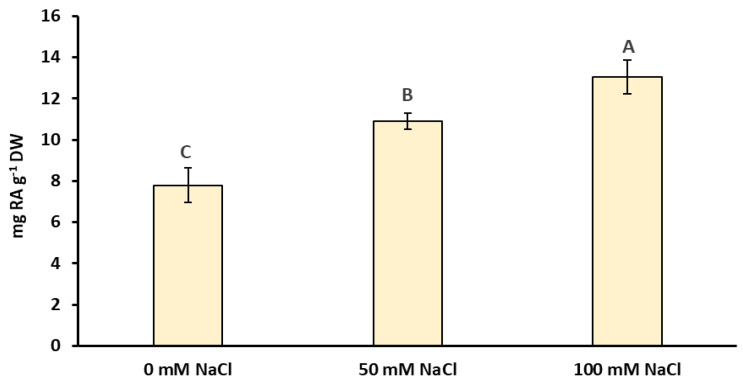
Effect of the NaCl application on the concentration of rosmarinic acid in *Melissa officinalis* shoots after 10 days of exposure. Data are means ± SD (*n* = 4). Means followed by different letters differ statistically significantly (*p* < 0.05, Tukey’s test).

Based on the UPLC-UV-MS analysis, 13 different phenolic acids, mainly hydroxycinnamic acid derivatives including a caffeic acid ester—rosmarinic acid and a caffeate trimer—lithospermic acid, were identified (Figure 3). These acids represented two characteristically high peaks in the chromatograms (Figure 4). All the chromatograms obtained from both the control and the NaCl-treated plants showed a similar phenolic profile, showing differences only in the quantities of each compound. No peaks from the new compounds were observed under the influence of NaCl (Figure 4). It was found that both salinity concentrations induced the accumulation of phenolic acids (Figure 4 and Figure 5). Quantitative determination of rosmarinic acid showed that its level increased by 40% and 67%, respectively, under the influence of 50 and 100 mM NaCl in relation to the control (Figure 5).

**Figure 6:**
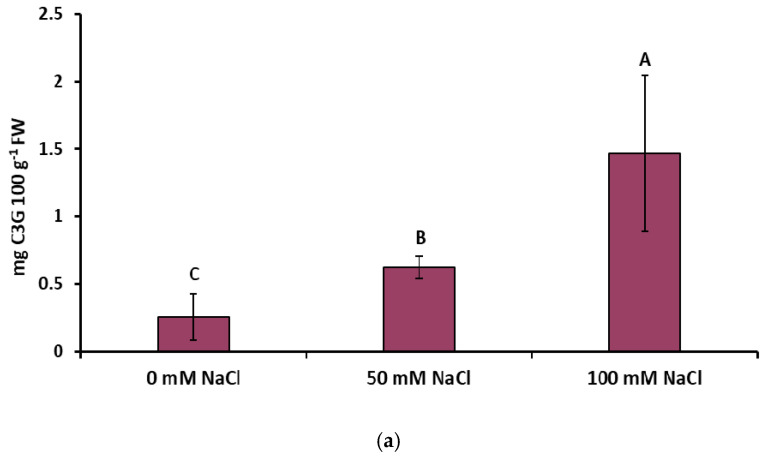

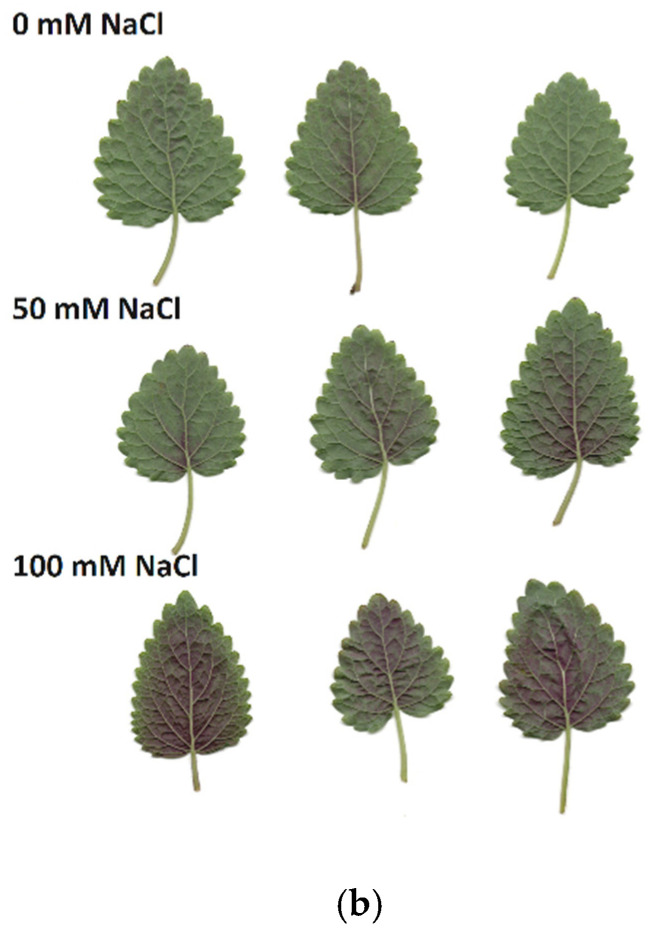
Effect of the NaCl concentration on (**a**) the foliar concentration of anthocyanins (as cyanidin 3-glycoside; C3G) and (**b**) pigmentation of the lower epidermis of *Melissa officinalis* leaves after 10 days of salt exposure. Data are means ± SD (*n* = 4). Means followed by different letters differ statistically significantly (*p* < 0.05, Tukey’s test).

The elicitation of the lemon balm with both concentrations of NaCl boosted the accumulation of anthocyanins in lemon balm leaves (Figure 6a), especially in the lower epidermis (Figure 6b). Along with the increase in salinity, over a two-fold or four-fold increase in the concentration of these compounds was found after the exposure to 50 mM NaCl or 100 mM NaCl, respectively.

**Figure 7:**
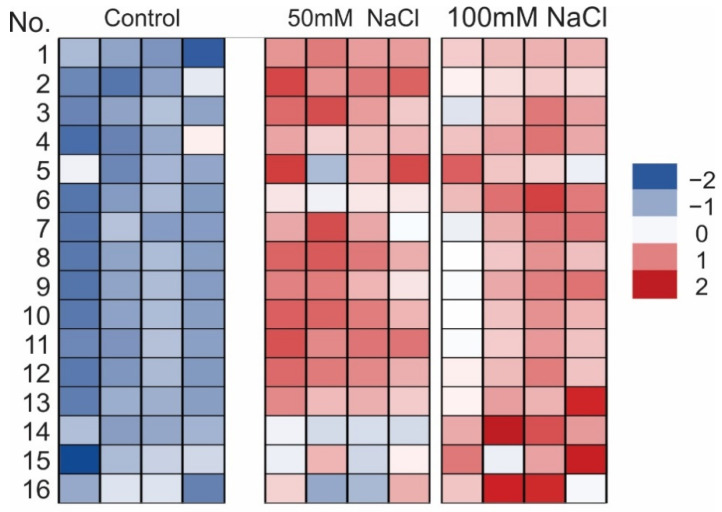
Heat map visualization of changes in the abundance of particular compounds shown in the rows for individual plant samples with different salinity levels (column). The colors range from dark blue (low abundance) to deep red (high abundance); number of compounds: (1) 3-(3,4-dihydroxyphenyl)-lactic acid; (2) caftaric acid; (3) fertaric acid; (4) caftaric acid hexoside; (5) rosmarinic acid hexoside; (6) rosmarinic acid; (7) salvianolic acid H/I (isomer); (8) salvianolic acid C derivative III; (9) lithospheric acid; (10) salvianolic acid C derivative III; (11) salvianolic acid C derivative IV; (12) sagecoumarin 2-hydroxy-3-(3,4-dihydroxyphenyl)-propanoide; (13) methyl lithospermic acid; (14) total anthocyanins; (15) soluble phenols; and (16) soluble flavonols.

The heat map (Figure 7) represents graphically the abundance of particular phenolic compounds across the experimental treatments. The standardized parameters are represented by colors (dark blue represents low value while dark red denotes high value). Four individuals are shown for each treatment. Additionally, the heat map also shows the aforementioned enhanced accumulation of anthocyanins, soluble phenols, and soluble flavonols in the NaCl treatments; however, this phenomenon was more notable at the higher concentration of salt (Figure 7). Moreover, the calculated mean contents of rosmarinic acid and anthocyanin per plant in the control plants were 4.6 mg and 7.7 ng, respectively, and increased to 6.6 mg and 33.9 ng per plant after eliciting with 100 mM NaCl. Meanwhile, TPC and TFC were at a similar level per plant in different treatments (Supplementary Table S1). The analysis of the influence of NaCl on the ability of the plant extracts to reduce DPPH radical revealed a significant increase in free radical scavenging activity (FRSA) only in the 100 mM NaCl treatment (Supplementary Figure S2).

## 3. Discussion

Salt-induced osmotic stress leads to numerous physiological disorders in plants, including water deficit, nutrient imbalance, membrane damage, hormonal imbalance, and oxidative damage [23]. For the above reasons, the biomass of lemon balm can decrease at various NaCl concentrations in the irrigation water used in our experiments (Figure 1a), which was also demonstrated by Khalid and Cai [21]. Similarly, in another medical species, i.e., sage, irrigated with a 100 mM NaCl solution, a significant reduction in growth parameters was demonstrated [24]. Nevertheless, at moderate levels, salt stress can also be an effective method of eliciting plant secondary metabolites [25].

Although the lemon balm biomass was significantly reduced under the influence of 100 mM but not 50 mM NaCl (Figure 1a), the analyzed parameters of chlorophyll *a* fluorescence did not indicate disturbances in photosynthesis in either of the NaCl treatments (Supplementary Figure S1). Also, the concentration of photosynthetic pigments was generally not negatively affected even in the 100 mM NaCl treatment. In the salinity conditions, the level of chlorophyll *b* was even increased (Figure 1a). Therefore, it seems that lemon balm may be more resistant to salinity than previously suggested by Bonacina et al. [20]. In contrast, in a study conducted by Safari et al. [22], a decrease in the content of chlorophyll under salt stress was found in this species. Studies conducted on sunflower have shown that the NaCl-induced reduction of chlorophyll level may be mainly a consequence of inhibited synthesis of 5-aminolaevulinic acid, a precursor of chlorophyll and, to a lesser extent, increased activity chlorophyll-degrading chlorophyllase [26].

However, in the context of the present research, a more interesting issue was the increase in the accumulation of bioactive substances under salinity. Besides essential oils, (poly)phenolic compounds with a broad spectrum of biological activities are the main group of secondary metabolites in the Lamiaceae family [27]. As shown by Ozarowski et al. [28], caffeic acid esters and glycosides of flavones are the major group of active metabolites in *M. officinalis*. Additionally, they indicated that the rosmarinic acid is a dominant active compound in this species. In our study, 13 caffeic acid derivatives were identified. Rosmarinic acid and lithospheric acid were the main phenolic acids identified in the extracts obtained from the shoots of the lemon balm (Figure 3 and Figure 4). It was found that the accumulation of different phenolic compounds, including caffeic acid derivatives, was clearly enhanced under salinity (Figure 4 and Figure 7). The concentration of rosmarinic acid was improved by 40–67% in comparison to the control (Figure 5). In turn, rosmarinic acid concentrations in five genotypes of *M. officinalis* were reduced by osmotic stress induced by drought, while the level of essential oils increased in some genotypes [29]. The phenomenon of an increasing level of (poly)phenolic compounds in response to various abiotic stress factors was observed before [30,31,32]. Although the mechanisms of (poly)phenol biosynthesis were not investigated here, ample evidence indicated that the phenylpropanoid pathway which generates a majority of compounds is activated by stress factors [33]. Previously, a positive correlation between salinity and phenolic compound concentrations was noted in *Thymus* species [34] or *Fagopyrum esculentum* [33]. In contrast, the content of phenolic compounds decreased in response to NaCl in broccoli [35] or lettuce [36], which indicates differential responses of plant species to salinity in relation to accumulation of (poly)phenolics.

Our recent studies on the application of a biotic elicitor chitosan lactate in basil and lemon balm showed that the foliar application of this compound effectively induced accumulation of phenolic compounds [37]. Here, the NaCl-induced elicitation generally had a positive effect on the level of all (poly)phenolics tested. However, the accumulation of TPC and TFC as well as FRSA was significantly improved only at the higher level of salinity (Figure 2a,b, Supplementary Figure S2). Much more effective elicitation of TPC was achieved in a study with *Mentha pulegium* [38], where a 3.5-fold increase in its level in leaf extracts was found in a 100 mM NaCl treatment. Unfortunately, a drastic reduction of plant biomass (approx. 60%) was recorded in these conditions. In turn, in another medicinal species belonging to the Lamiaceae family, i.e., *Salvia macrosiphon*, the content of TPC decreased under salinity, but increased leaf antioxidant capacity was demonstrated [39]. In a mangrove halophyte *Aegiceras corniculatum* exposed to 250 mM NaCl, the levels of (poly)phenols increased more than twofold, which may indicate their protective role under salt stress [40].

In this study, the accumulation of anthocyanins increased several fold in response to the salinity stress (Figure 6a). Changes in the anthocyanin content were also visible as differences in the color of the lower leaf epidermis (Figure 6b). Analogous results were obtained in a study conducted by Jahantigh et al. [41] on hyssop (Lamiaceae), which indicated a significant increase in the level of both TPC and anthocyanins after application of saline water (2–10 mS cm^−1^). The enhanced accumulation of anthocyanins in plants exposed to salinity has been largely documented (e.g., [42,43]). The protective role of anthocyanins under salt stress includes their antioxidant capacity in response to ROS overproduction triggered by imbalance in Na^+^/K^+^ ion homeostasis [44]. Moreover, these compounds can be involved in reduction of the leaf osmotic potential, which is directly related to improved plant water status under osmotic stress, suggesting that accumulation of anthocyanins can be a quick and beneficial plant response to salt stress [45]. It should also be emphasized that the increase in the concentration of anthocyanins and rosmarinic acid, both with well-known health-promoting properties [46,47], overcompensates the decrease in plant biomass under salt stress (Supplementary Table S1).

## 4. Materials and Methods

### 4.1. Plant Material and Growth Conditions

Twenty seeds of lemon balm (*Melissa officinalis* L.) were sown in 12 pots with a capacity of 0.5 L and allowed to germinate. The soil substrate was the universal organic COMPO BIO substrate with the addition of guano and compost produced on the basis of high peat, with pH = 5.0–7.0. Seed germination as well as further growth and development of plants took place in a phytotron room equipped with air conditioning and fluorescent lamps. The plants were grown at photosynthetic photon flux density (PPFD) at the level of the tops of the plants of 170–200 µmol m^−2^ s^−1^, 14-h photoperiod, at day/night temperature of 27/23 °C, and 60–65% relative humidity. Due to the uneven germination of the seeds, the plants were thinned after about 21 days, and 12 plants were left in each pot.

The experiment was differentiated 36 days after sowing (DAS), and three treatments were established. Control plants (in four pots) were watered with 50 mL of distilled water, while the other plants were treated with the same volume of NaCl solutions with a concentration of 50 mM (5.38 mS cm^−^^1^; four pots) or 100 mM (10.16 mS cm^−1^; four pots). A similar irrigation scheme was applied after the next 2, 5, and 7 days. In total, 200 mL of solutions with different NaCl concentrations (0, 50, or 100 mM) were introduced into each pot.

After 10 days of lemon balm growth in the different experimental conditions (10 days after the application of the first dose of NaCl), the biometric (biomass of shoots), physiological (photosynthetic pigments, chlorophyll fluorescence), and phytochemical (concentration of total phenolics, soluble flavonoids, and anthocyanins, quantitative analysis of rosmarinic acid, identification of phenolic acids, free radical scavenging activity) parameters were determined.

### 4.2. Determination of Biomass and Physiological Parameters

The chlorophyll *a* fluorescence parameters (the maximal, F_m_; and minimal, F_0_ possible level of fluorescence; the maximum quantum yield of PS II, F_v_/F_m_; where F_v_ = F_m_ − F_0_) were measured on the fourth pair (from the top) of dark-adapted (15 min) leaves. Ten different individuals per treatment were randomly selected, and a chlorophyll fluorimeter (Handy PEA, Hansatech Instruments, Pentney, UK) was used for fluorescence determinations.

The concentration of photosynthetic pigments (chlorophylls and carotenoids) was measured using the method proposed by Lichtenthaler and Wellburn [48]. The leaf samples were taken from the fourth pair from the top, homogenized in 80% (*v*/*v*) acetone, and filtered. Absorbance of the extracts was measured at 663 nm, 646 nm, and 470 nm (Cecil CE 9500, Cecil Instruments, Cambridge, UK).

Then, the aboveground parts of four plants from each pot were collected (16 plants from each treatment), and their fresh weight (FW) was determined using a laboratory balance.

### 4.3. Preparation of Extracts for Determination of Total Phenolics, Flavonols, Phenolic Acids, and Free Radical Scavenging Activity

Plant material from each pot dried at 55 °C was used for preparation of extracts. Samples were extracted with 5 mL of 80% (*v*/*v*) methanol at room temperature for 1/2 h in an ultrasonic bath. The extracts were centrifuged (10 min at 4500× *g*), and the clear supernatant was used for further analysis.

### 4.4. Analysis of Phenolic Compounds and Free Radical Scavenging Activity

The total phenolic content (TPC) was determined using the Folin-Ciocalteu phenol reagent, following the method proposed by Wang [49] with slight modifications. The test sample (0.1 mL) was mixed with 1.9 mL of re-distilled water and 1 mL of Folin-Ciocalteu’s reagent. After 5 min, 3 mL of a saturated Na_2_CO_3_ solution were added. The reaction mixture was kept at 40 °C in the dark for 30 min. Absorbance was measured at 756 nm (Cecil CE 9500, Cecil Instruments, Cambridge, UK) against the reagent blank. The concentration of phenolic compounds was calculated as gallic acid equivalents (GAE) per g of dry plant material.

Soluble flavonols were determined with the colorimetric method as a complex with aluminum ions [50]. The absorbance was read at 425 nm (Cecil CE 9500, Cecil Instruments, Cambridge, UK) after 30 min of dark incubation of the test sample (0.3 mL) with 0.75 mL of a 2% AlCl_3_ methanolic solution (*w*/*v*) and 0.45 mL of 80% methanol (*v*/*v*) against the reagent blank. The concentration of flavonoids was calculated as rutin equivalents (RE) per g of dry plant material.

The accumulation of anthocyanins in the fresh leaves (fourth pair from the top) was determined using the method described previously [51]. Anthocyanins were extracted by maceration of leaf samples in a methanol:HCl solution (99:1, *v*/*v*). The extracts were centrifuged, and their absorbances were read at 527 nm and 652 nm (Cecil CE 9500, Cecil Instruments, Cambridge, UK). The concentration of anthocyanins was calculated using the extinction coefficient (ε = 29,600 M^−1^ cm^−1^) for cyanidin 3-glycoside (C3G).

The methanol extracts were analyzed using an Agilent Technology 1290 Infinity Series II ultra-high performance liquid chromatograph (UHPLC) equipped with a DAD detector and an Agilent 6224 ESI/TOF mass detector (Agilent Technologies, Santa Clara, CA, USA). The ion source operating parameters were as follows: drying gas temperature 325 °C, drying gas flow 5.0 l min^−1^, and capillary voltage 3500 V. Ions were acquired in the range from 100 to 1050 m/z in the negative ion mode. Agilent Technologies Mass Hunter software version 10.00 00 (Agilent Technologies, Santa Clara, CA, USA) was used for data acquisition and data analysis. The separation was performed with a method described previously [52]. Briefly, an RP18e LiChrosper 100 column (Merck, Darmstadt, Germany) (25 cm × 4.9 mm i.d., 5 µm particle size) was used to separate phenolic acids. The linear gradient from 5% to 20% of acetonitrile in water within 45 min was applied. The flow rate was 1.0 mL/min. The column temperature was set at 25 °C. The identity and quantification of rosmarinic acid was performed based on comparison with a standard compound.

The identification of 13 different phenolic acids was possible using UPLC-TOF/MS analysis (Figure 3), as in Ozarowski et al. [28] and Barros et al. [53].

The free radical scavenging activity (FRSA) was determined using DPPH (1,1-diphenyl-2-picrylhydrazyl) stable radical. The test sample (50 µL) was added to 2 mL of a DPPH solution (200 µmol L^−1^). A total of 50 µL of 80% methanol (*v*/*v*) was added to the control sample. The absorbance of the control and test samples was determined at 517 nm (Cecil CE 9500, Cecil Instruments, Cambridge, UK) after 15 min of dark incubation. Results are reported as percentage DPPH reduction by the plant extracts.

### 4.5. Statistical Analyses

The data were subjected to one-way ANOVA followed by a Tukey’s post-hoc test (*p* < 0.05). Statistica ver. 13.3 software (TIBCO Software Inc. 2017, Palo Alto, CA, USA) was used for the statistical analysis. The heat map was constructed based on standardized data with Microsoft Excel (2010).

## 5. Conclusions

Our study demonstrates that NaCl irrigation functions as an activator of accumulation of (poly)phenolics. These results show, for the first time, enhanced accumulation of hydroxycinnamic acid derivatives in lemon balm under salinity. The increase in anthocyanin concentration was several fold. It is worth emphasizing that the biomass of the aboveground parts did not decrease significantly under the influence of 50 mM NaCl, and its reduction in the 100 mM NaCl treatment was significant but not very large. In the salt treatments, there were no significant disturbances in photosynthesis parameters and the content of photosynthetic pigments. Therefore, NaCl is a cheap and efficient abiotic elicitor that may be potentially used in elicitation of phenolic metabolites in lemon balm under pot cultivation. However, we do not recommend this elicitation method in field conditions due to the limited possibility of later removal of NaCl from the soil.

## Data Availability

The data presented in this study are available on request from the corresponding author.

## Supplementary Materials

Supplementary Materials can be found at https://www.mdpi.com/article/10.3390/ijms22136844/s1.
