# Supplementary material for: NaCl-Induced Elicitation Alters Physiology and Increases Accumulation of Phenolic Compounds in Melissa officinalis L
Source: Int J Mol Sci. 2021 Jun 25;22(13):6844. doi: 10.3390/ijms22136844 (PMC8268150; doi:10.3390/ijms22136844)
Supplement: Supplementary file 1 [file ijms-22-06844-s001.zip › ijms-1265167-supplementary.pdf]

SUPPLEMENTARY MATERIALS

NaCl-induced Elicitation Alters Physiology and Increases Accumulation of Phenolic Compounds in *Melissa officinalis* L.

Barbara Hawrylak-Nowak [1]*, Sławomir Dresler [2], Maria Stasińska-Jakubas [1], Magdalena Wójciak [2], Ireneusz Sowa [2], and Renata Matraszek-Gawron [1]

[1] Department of Botany and Plant Physiology, Faculty of Environmental Biology, University of Life Sciences in Lublin, Akademicka 15, 20-950 Lublin, Poland; barbara.nowak@up.lublin.pl (B.H.N); jakubas.ms@gmail.com (M.S.J); renata.matraszek@up.lublin.pl (R.M.G)
[2] Department of Analytical Chemistry, Medical University of Lublin, Chodźki 4a, 20-093 Lublin, Poland; slawomir.dresler@umlub.pl (S.D.); magdalenawojciak@umlub.pl (M.W.); i.sowa@umlub.pl (I.S.)
* Correspondence: barbara.nowak@up.lublin.pl

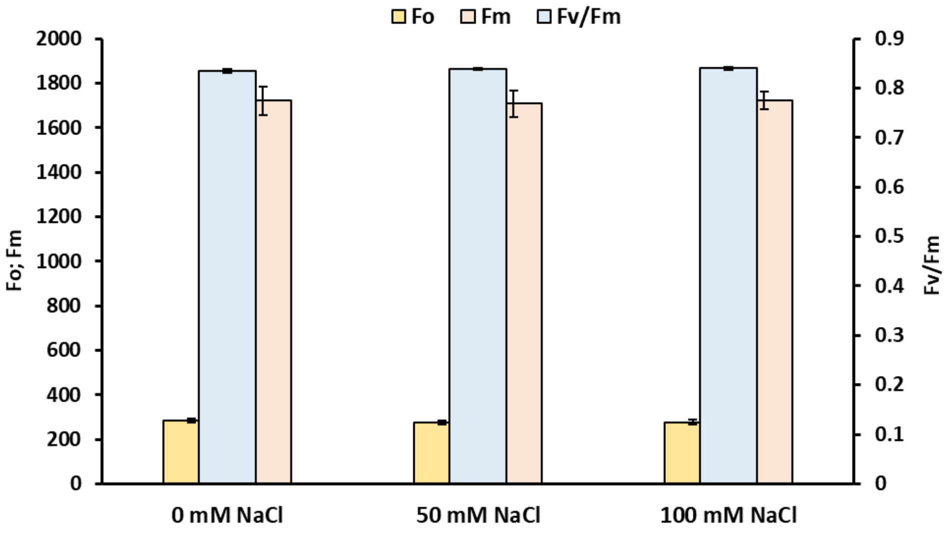

Supplementary Figure S1. Effect of NaCl application on the selected parameters of chlorophyll *a* fluorescence in *Melissa officinalis* after 10 days of exposure. Data are means ±SD (n=10). The absence of letters means that there are no significant differences between treatments (p<0.05, Tukey's test).

Supplementary Table S1. Effect of NaCl application on the content of selected phenolic compounds per plant of *Melissa officinalis* after 10 days of exposure.

Content of phenolic compounds	NaCl concentration		
	0 mM NaCl	50 mM NaCl	100 mM NaCl
soluble phenols (mg per plant)	22.18	22.11	22.19
soluble flavonols (mg per plant)	9.90	9.74	10.48
rosmarinic acid (mg per plant)	4.60	5.88	6.63
anthocyanins (ng per plant)	7.71	16.00	33.90

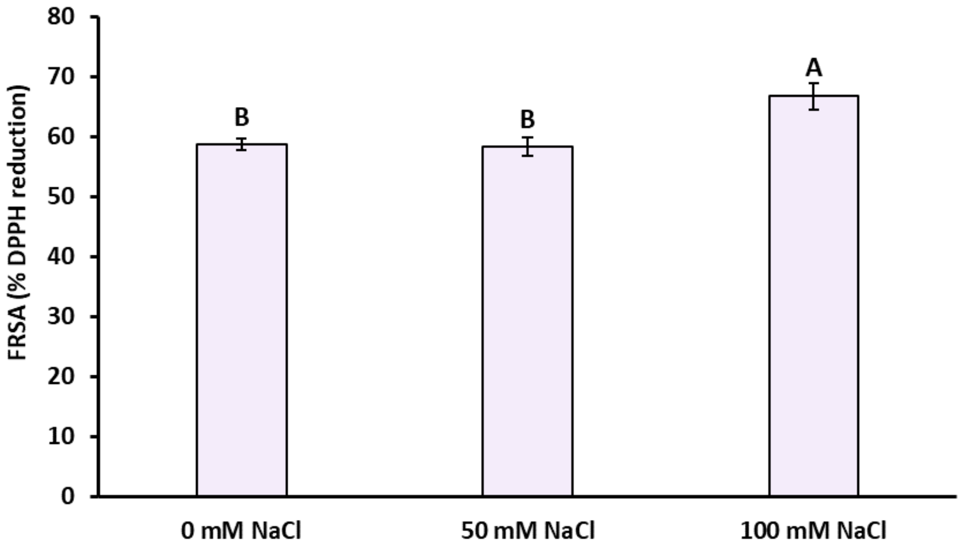

Supplementary Figure S2. Free radical scavenging activity (FRSA) of extracts of *Melissa officinalis* grown for 10 days at different NaCl treatments. Data are means ±SD (n=4). Means followed by different letters differ statistically significantly ($p<0.05$, Tukey's test).
